# Establishment of *Iris laevigata* Tissue Culture Using Hypocotyl and Root Explants

**DOI:** 10.3390/plants14172733

**Published:** 2025-09-02

**Authors:** Nuo Xu, Haijing Fu, Yujia Liu, Aruna Kilaru, Jyoti R. Behera, Ling Wang

**Affiliations:** 1 College of Landscape Architecture, Northeast Forestry University, Harbin 150040, China; xunuo@nefu.edu.cn (N.X.); 13654632858@163.com (H.F.); liuyujia6209@126.com (Y.L.); 2 Department of Biological Sciences, East Tennessee State University, Johnson City, TN 37614, USA; kilaru@etsu.edu (A.K.); behera@mail.etsu.edu (J.R.B.)

**Keywords:** *Iris laevigata*, tissue culture, regeneration, callus, phytohormone, plant growth regulator

## Abstract

*Iris laevigata* is an ornamental plant and so its wild genetic resources need to be protected. However, traditional inefficient propagation limits its landscape applications. In this study, we assessed the effects of phytohormones on growth of *I. laevigata* at various culture stages using roots and hypocotyls as explants and established an efficient micropropagation system. The highest callus induction of hypocotyl (75.0%) was obtained using Murashige and Skoog medium containing 6-benzylaminopurine (6-BA), 0.5 mg L^−1^ + 2,4-dichlorophenoxyacetic acid (2,4-D), 1.0 mg L^−1^ + 1-naphthylacetic acid (NAA), and 0.4 mg L^−1^. Similarly, the highest callus induction (73.3%) of roots was achieved with 6-BA 0.5 mg L^−1^ + 2,4-D 0.5 mg L^−1^ + NAA 0.4 mg L^−1^. The calli induced from hypocotyl and root tissues achieved 39.7% and 49.5% adventitious shoot induction on a medium containing indole-3-butyric acid (IBA) 0.5 mg L^−1^ + 6-BA 1.5 mg L^−1^ + NAA 1.0 mg L^−1^ and 6-BA 2.0 mg L^−1^ + NAA 0.4 mg L^−1^ + kinetin (KT) 1.0 mg L^−1^, respectively. The rooting of adventitious shoots reached 93.3% in the medium supplemented with NAA 0.2 mg L^−1^. The survival of regenerated plants reached 90.0% after being transplanted into soil. This study provides an efficient and reliable propagation method for *I. laevigata* for landscape applications and the preservation of wild genetic material.

## 1. Introduction

*Iris laevigata*, commonly referred to as Japanese water iris, is a perennial herbaceous flowering plant of the family Iridaceae, which has gained increasing attention in recent years. In the wild, this aquatic plant is primarily found alongside rivers, marshes, and wetlands in China, Japan, South Korea, and Russia [1]. Due to its gorgeous blue flowers, which are larger than many *Iris* species, it is regarded as a plant with high ornamental value. It has a long history of cultivation in northeastern China and many renowned botanical gardens around the world [2]. In addition to its landscape applications, *I. laevigata* is also the parent of many popular horticultural varieties; therefore, it is considered to be a significant genetic resource for *Iris* breeding. Because of its native ecological origin, it exhibits a strong tolerance to cold and waterlogged conditions. These physiological characteristics make it a valuable germplasm resource for developing new ornamental *Iris* cultivars, which can be adapted to waterlogged and cold conditions in different landscaping areas [3,4,5]. The increasing loss and damage of its habitats, however, pose a critical challenge to the survival of native wild populations. As such, it has been defined as one of the second-grade endangered species in South Korea [6].

Traditionally, *Iris* reproduces either by bulbs or the splitting of rhizomes. However, vegetative propagation through bulbs or rhizome splitting is generally slower compared to growing from seeds. It may take a few years for divided clumps to mature and produce a substantial number of flowers. Propagation by seed can also be challenging as seeds require a cold stratification period and plants can often take several years to reach maturity from a seed. Furthermore, due to the limitations of cross-pollination and seed germination, sexual reproduction cannot achieve efficient large-scale propagation [7,8]. Both sexual and asexual propagation can also be limited by the season and the state of plant growth.

Micropropagation or tissue culture is a common propagation method used to generate new offspring rapidly for ornamental horticulture applications. It is also a viable strategy for increasing the number of individuals ex situ to save endangered species [9]. Moreover, the establishment of a callus formation and whole-plant regeneration system is also one of the basic requirements for genetically engineering and further developing plant genetic resources [10]. Previous studies have shown that it is more difficult to culture Iridaceae in vitro compared to other monocotyledonous plants such as Amaryllidaceae, Araceae, and Liliaceae [7,11,12]. The cell differentiation of monocotyledons, including *Iris*, starts early and proceeds rapidly, limiting the explant source to only the meristematic region and adjacent tissues. This hinders the establishment of in vitro regeneration systems for *Iris* [7]. The composition of plant growth regulators (PGRs) in the growth medium is also one of the most important factors affecting in vitro propagation. For decades, the in vitro propagation of several *Iris* species with high ornamental value or danger of extinction (*I. germanica*, *I. ensata*, *I. pallida*, *I. pseudacorus*, *I. pumila*, *I. setosa*, *I. sibirica*, *I. versicolor*, etc.) has been achieved using anthers, bulbs, flowers, inflorescences, leaves, ovaries, roots, twin scales, and shoots [7,13,14]. However, a reliable system for callus formation and regeneration in *I. laevigata* is currently lacking. Therefore, our objective was to develop an efficient callus formation and regeneration system for *I. laevigata* to enhance its horticultural and molecular applications and contribute to the conservation of this natural resource.

In this study, we developed suitable culture media containing PGRs and assessed their suitability for callus induction and adventitious organ induction using two explants, hypocotyl and root. Additionally, the effects of PGRs on different stages of plant regeneration were also studied. The survival efficiency of regenerated plants was used to evaluate and develop an efficient and reliable callus regeneration system for *I. laevigata*.

## 2. Results

### 2.1. Effect of Different PGR Concentrations on I. laevigata Hypocotyl Callus Induction

**Figure 1:**
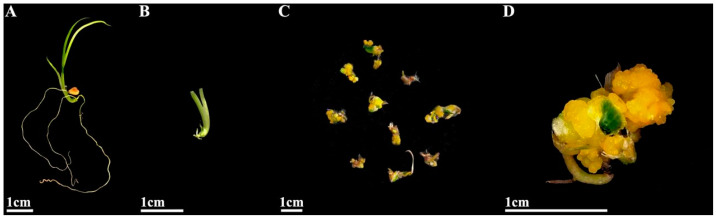
Callus induction from hypocotyl explants. (**A**) Germinated seeds at 20 days; (**B**) hypocotyls as explants with roots removed and leaves pruned; (**C**) calli induced from hypocotyls at 45 days; (**D**) calli observed under stereomicroscope.

**Figure 2:**
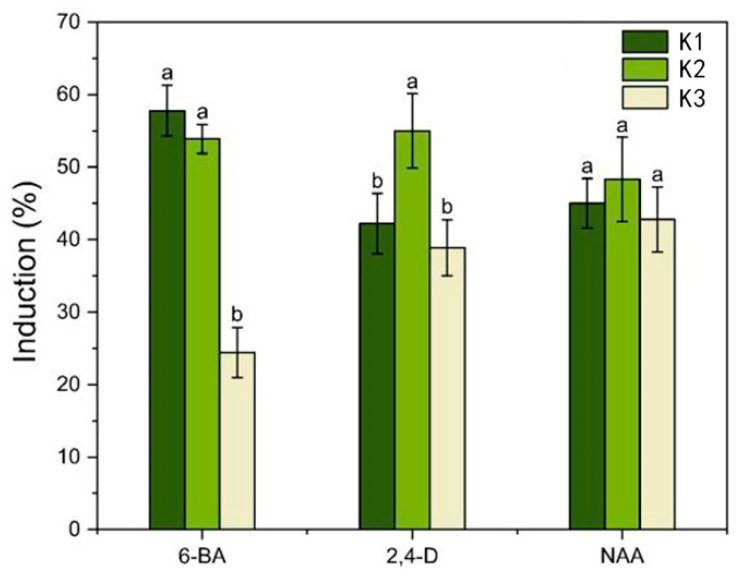
Analysis of the effect of plant growth regulators (PGRs) on the hypocotyl callus induction. Each value represents the mean ± SEM of three independent experiments. Different letters of significance are indicated for each treatment at *p* < 0.05 as determined by three-way analysis of variance (ANOVA) with Duncan’s post-test. K1, K2, and K3 represent the average induction rates of the same factor at different levels, with concentrations increasing from low to high. The specific data values are shown in Table S2.

Sterile *I. laevigata* seedling hypocotyls were used as explants and placed flat on MS medium with different PGR concentrations to induce calli development. The induction and appearance of calli were recorded after 45 days (Figure 1C,D). As the 6-BA concentration was increased from 0.5 mg L^−1^ to 2.0 mg L^−1^, the mean induction value initially increased significantly, but at higher concentrations, it began to decrease (Figure 2). The mean induction value exhibited a significant initial increase followed by a subsequent decrease as the concentration of 2,4-D increased from 0.5 mg L^−1^ to 1.5 mg L^−1^ (Figure 2). Furthermore, according to the range analysis (Table S1), the influence of the PGRs on hypocotyl callus induction was ranked as 6-BA > 2,4-D > NAA. The analysis of variance (Table S1) indicated that 6-BA and 2,4-D were significant factors involved in the hypocotyl induction (*p* < 0.05) while NAA had relatively little influence (*p* > 0.05). Therefore, multiple comparisons were performed on different levels of 6-BA and 2,4-D, and the results are shown in Table S2. The induction rate was highest when the concentration of 6-BA was at K1; thus, the optimal concentration of 6-BA is 0.5 mg L^−1^. Similarly, the induction rate was highest when the concentration of 2,4-D was K2; thus, the optimal concentration of 2,4-D is 1.0 mg L^−1^. The rate of callus induction with treatment 2 was 75%, and the condition of the induced calli was good (Table S2). Therefore, the optimal medium for *I. laevigata* hypocotyl callus induction was 6-BA 0.5 mg L^−1^ + 2,4-D 1.0 mg L^−1^ + NAA 0.4 mg L^−1^ + MS + sucrose 30 g L^−1^ + agar 7 g L^−1^.

### 2.2. Effects of Different PGR Concentrations on I. laevigata Root Callus Induction

**Figure 3:**
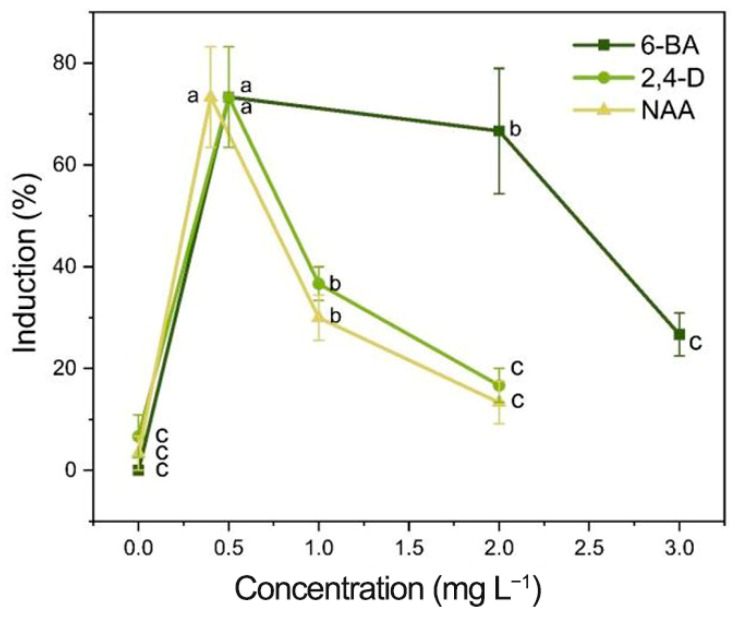
Effects of different concentrations of PGRs on the induction of root calli. Each value represents the mean ± SEM of three independent experiments. Different letters of significance are indicated for each treatment at *p* < 0.05 as determined by three-way analysis of variance (ANOVA) with Duncan’s post-test.

**Figure 4:**
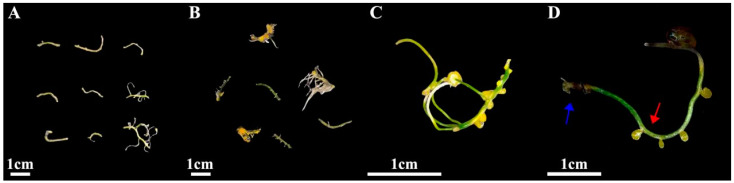
(**A**) Callus induction from root after 1 day; (**B**) callus induction from root after 45 days; (**C**) root-induced calli under stereomicroscope; (**D**) the incision site where the secondary roots were cut off (red arrow) and the incision site on the adventitious root (blue arrow).

To determine the impact of the three PGRs on *I. laevigata* root callus induction, two types of *I. laevigata* root explants were investigated. One set of root explants had no secondary roots, while the second set of root explants had secondary roots that were removed. The root explant segments without secondary roots did not produce any calli, irrespective of the phytohormone concentration in the growth medium. Therefore, we focused only on the induction of calli induction in root explants with secondary roots that were originally attached but had been removed prior to induction (Figure 3 and Figure 4; Table S3).

The induction process is shown in Figure 4, where new callus tissue grows at the incision site on the root (Figure 4C,D). The induction and appearance of calli varied greatly with the PGRs tested (Table S3). When the concentrations of 6-BA and NAA remained constant, the root callus induction increased as the 2,4-D concentration increased, resulting in a single peak curve (Table S3; Figure 3). A single peak curve was also observed when the 6-BA and 2,4-D concentrations remained constant, and the NAA concentration increased (Table S3; Figure 3). The highest callus induction in the root explants was produced when the media contained 2,4-D at 0.5 mg L^−1^ and NAA at 0.4 mg L^−1^ with either 0.5 or 2.0 mg L^−1^ 6-BA (Table S3; Figure 3). When the media did not contain 6-BA (treatment 1, 0 mg L^−1^), there was no calli induction (0%) for the root explants (Table S3). When either 2,4-D or NAA were absent from the media (treatments 5, 0 mg L^−1^; and treatments 8, 0 mg L^−1^), the callus induction was very low, 6.7% and 3.3%, respectively. As a result, although 6-BA had the most significant impact on root callus induction, the presence of low levels of 2,4-D (0.5 mg L^−1^) and NAA (0.4 mg L^−1^) were essential for maximizing callus induction (Table S3). Therefore, to produce maximum callus induction with *I. laevigata* root explants, the medium used must contain the three PGRs (6-BA, 2,4-D, and NAA). Although the levels of callus induction in treatment 2 (6-BA 0.5 mg L^−1^ + 2,4-D 0.5 mg L^−1^ + NAA 0.4 mg L^−1^) and treatment 3 (6-BA 2.0 mg L^−1^ + 2,4-D 0.5 mg L^−1^ + NAA 0.4 mg L^−1^) were not significantly different (73.3% and 66.7%), treatment 2 required less 6-BA and produced numerically greater callus inductions at 45 d (Table S3). As a result, we choose the following medium formulation for *I. laevigata* root callus induction: 6-BA 0.5 mg L^−1^ + 2,4-D 0.5 mg L^−1^ + NAA 0.4 mg L^−1^ + MS + sucrose 30 g L^−1^ + agar 7 g L^−1^.

### 2.3. Effects of Different PGRs and Their Concentrations on Adventitious Shoot Induction of Hypocotyl and Root Calli

**Figure 5:**
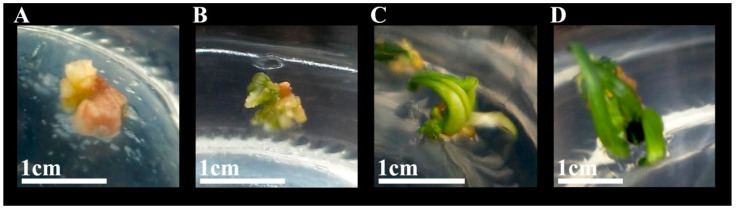
Process of adventitious induction of hypocotyl-induced calli from shoots. (**A**) Without adding IBA to the media, hypocotyl-induced calli were browning, and (**B**) not completely browned and no adventitious shoots were produced. When 0.5 mg L^−1^ of IBA was added to the medium, the adventitious shoots grew green and strong after (**C**) 30 days and (**D**) 40 days.

Three media formulations were evaluated to determine their impact on the adventitious induction of hypocotyl calli from shoots (Table S4). The highest induction (39.7%) was achieved after 60 days with a medium containing IBA 0.5 mg L^−1^ + 6-BA 1.5 mg L^−1^ + NAA 1.0 mg L^−1^ + MS + sucrose 30 g L^−1^ + agar 8 g L^−1^ (Table S4). These results confirm that IBA significantly promotes the adventitious induction of hypocotyl calli from shoots. While no adventitious shoots were produced in the media without IBA (Figure 5A,B), the adventitious shoots were green and strong when 0.5 mg L^−1^ IBA was added (Figure 5C,D).

**Figure 6:**
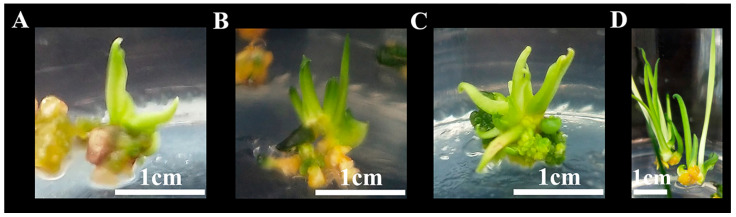
Adventitious shoot induction process of root induced calli. Adventitious shoots were induced for (**A**) 20 days, (**B**) 25 days, (**C**) 35 days, and (**D**) 50 days. parts.

Root-induced calli were placed into the adventitious shoot induction media, which included various concentrations of 6-BA, NAA, and KT, and were cultured for 60 days (Table S5). The maximum adventitious shoot induction for root-induced calli was 49.5%, which was achieved with treatment 7 (6-BA 2.0 mg L^−1^ + NAA 0.4 mg L^−1^ + KT 1.0 mg L^−1^ + MS + sucrose 30 g L^−1^ + agar 8 g L^−1^). The calli produced were green with healthy adventitious shoots (Figure 6).

**Figure 7:**
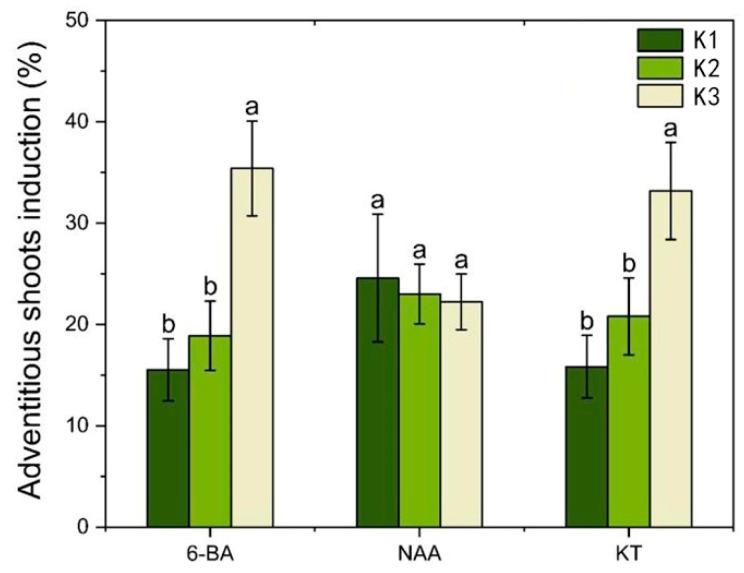
Analysis of the effect of PGRs on the inducti9on of adventitious shoots of root induced calli. Each value represents the mean ± SEM of three independent experiments. Different lowercase letters in the same column indicate significant differences at *p* < 0.05 as determined by three-way analysis of variance (ANOVA) with Duncan’s post-test. K1, K2, and K3 represent the average induction rates of the same factor at different levels, with concentrations increasing from low to high. The specific data values are shown in Table S5.

With the increase in 6-BA concentration, the average differentiation rate increased; with the increase in NAA concentration, the mean differentiation rate decreased; with the increase in KT concentration, the average differentiation rate increased (Figure 7). Combined with the magnitude of the *p*-value in Table S6 and the range analysis (Table S5), the influence of each factor was ranked as 6-BA > KT > NAA. The analysis of variance indicated that both 6-BA and KT had a significant impact on the adventitious shoot induction (*p* < 0.05), while NAA had relatively little influence (*p* > 0.05) (Table S6). Therefore, it is proposed that within the concentration range of 0.4–0.8 mg L^−1^, NAA can effectively support the induction of adventitious shoots from a callus induced by a root. When the concentration of 6-BA was at the K3 level, the rate of adventitious shoot induction reached its highest point, so the optimal concentration of 6-BA is 2.0 mg L^−1^. Similarly, when the concentration of KT was at the K3 level, the rate of adventitious shoot callus reached its highest point, so the optimal concentration of KT is 1.0 mg L^−1^. Additionally, treatment 7 showed the highest rate of adventitious shoot callus (49.5%) and the best growth conditions (Table S5).

### 2.4. Effect of Different PGR Concentrations on Root Induction of Adventitious Shoots

**Figure 8:**
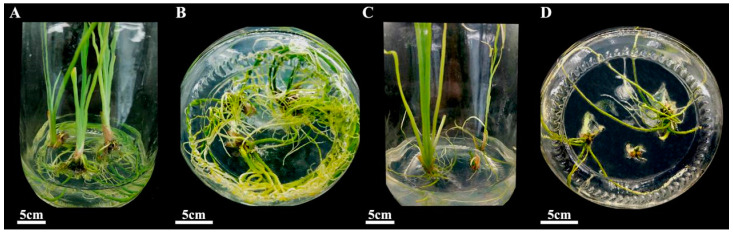
Roots induced by the medium supplemented with NAA or IBA for 40 days. (**A**) Adventitious shoots grew vigorously in the medium supplemented with NAA; (**B**) several roots were induced from adventitious shoots with NAA; (**C**) adventitious shoots grew weakly in the medium supplemented with IBA; (**D**) few roots were induced from adventitious shoots with IBA.

After 30 days of culturing, adventitious shoots were easily rooted in the MS medium supplemented with different concentrations of NAA or IBA. A rate of about 83.3% rooting was achieved in all combinations of media tested, with no significant difference among them (Table S7). However, the rooting coefficient varied from 3.13 to 8.12; NAA was more effective in improving the rooting coefficient than IBA. Also, the roots were stronger when NAA was added to the rooting medium (Figure 8), which was vital to the survival of plantlets. As such, NAA is more suitable for the induction of *I. laevigata* roots.

Among the three NAA concentrations,1.0 mg L^−1^ NAA showed the highest rooting coefficient, but the rate of rooting was only 83.3%, and the root growth was slow; adding 0.2 mg L^−1^ NAA let to the highest rate of rooting (93.3%) and a relatively high rooting coefficient (7.17); the roots also grew thicker and faster. Therefore, the best rooting medium is NAA 0.2 mg L^−1^ + MS + sucrose 30 g L^−1^ + agar 7 g L^−1^.

### 2.5. Acclimatization and Transplanting of Plantlets

**Figure 9:**
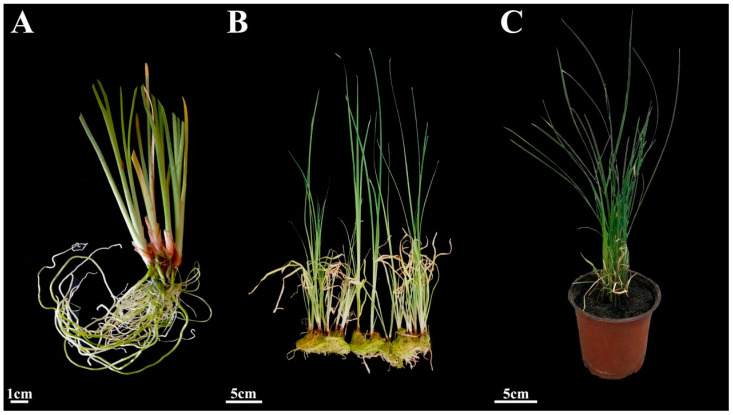
Acclimatization and transplanting of plantlets. (**A**) Regenerated plants after 3 days of acclimation. (**B**) Regenerated plants after 20 days of hydroponics. (**C**) Plants cultured in a greenhouse for 30 days.

The regenerated plantlets were acclimatized in water and then transplanted to potting soil (Figure 9). The survival of potted plantlets reached 90.0% after being cultured for 30 days in the greenhouse, and the plantlets grew vigorously.

## 3. Discussion

The source of explants has a decisive influence on the ability and efficiency of in vitro regeneration. Leaf bases, floral organs, shoots, and roots are often used for callus regeneration in *Iris* species, and the success of induction varied greatly from 39.1% to 80.9% [7,8,15,16]. The two explants used here, hypocotyls and roots, showed no significant difference in callus induction (75.0% and 73.3%, respectively) in the current study (Tables S2 and S3). Since mature seeds are easy to store, and can be used throughout the year, the sterile explants can be easily obtained from germinating seeds. Although the induction of calli with hypocotyls was not previously reported, this study unequivocally demonstrates that they can be used as explants to induce calli in *Iris* plants.

In this study, two types of roots were used as explants; root explants with no secondary roots (Type I), and the root explants with secondary roots that had been removed (Type II). Calli were observed to develop from the secondary root excise locations on the Type II explants (Figure 4), while no calli were induced from the Type I explants. Earlier reports indicated that callus formation induced by the roots of three *Iris* species predominantly originated from lateral root primordia, without specifying the cellular origin as being pericycle cells [11]. Our study showed that calli developed from the incision sites where the secondary roots were cut off (Figure 4) rather than the incision sites of adventitious roots (Figure 4) because there were active lateral meristems at site of the red arrow (Figure 4). Type I explants could not induce calli possibly because the pericycle had not dedifferentiated into actively dividing cells of lateral root primordia. No calli were induced at the blue arrow site on the Type II or Type I explants, indicating that the undifferentiated pericycle cells cannot directly produce calli. It seems that the source of explants for *I. laevigata* is extremely restrictive, and even using the root tips of sterile seedlings grown for 30 days cannot induce calli.

The composition of PGRs play an important role in plant regeneration [17,18]. The exogenous application of auxin and cytokinin induced calli in various plant species [19]. Previous reports emphasized the importance of exogenous 2,4-D in the regeneration of some *Iris* species [7,16]. It is considered to be the most effective auxin for callus induction [10], but it is usually required to prohibit or reduce shoot regeneration [20]. When root segments were used to induce calli, the induction was very low when only one auxin (2,4-D or NAA) was used. The combination of the three PRGs (6-BA, 2,4-D, and NAA) was more conducive to callus induction than any combination of two PRGs. The addition of another auxin to the induction medium contributes to the morphogenesis of some *Iris* species. Moreover, different types of auxin lead to different morphogenesis directions [21]. Among the three media we tested for the adventitious induction of hypocotyl shoot calli, treatment 2 (IBA 0.5 mg L^−1^, 6-BA 1.0 mg L^−1^, and NAA 0.4 mg L^−1^) was more effective than treatment 1 (6-BA 1.0 mg L^−1^ and NAA 0.4 mg L^−1^, Table S4). However, we could not determine whether this was due to the characteristics of IBA or the change in the ratio of cytokinin/auxin. In addition, our observations suggest that the induction of *I. laevigata* calli is dependent on the exogenous application of 6-BA. Hormone 6-BA (*p* < 0.05) was the most significant factor that affected the induction of hypocotyl and root calli, which may be due to the lack of endogenous cytokinin. In addition, both cytokinins (6-BA and KT) had significant effects on the induction of adventitious shoots (*p* < 0.05). We deduce that the exogenous cytokinins are more important than exogenous auxins in the in vitro regeneration of *I. laevigata*. A similar conclusion was reported for the propagation of *I. ensata* [20].

## 4. Materials and Methods

### 4.1. Plant Material

The seeds of *I. laevigata* were collected at the experimental nursery of Northeast Forestry University (126°64′ E, 45°72′ N, Harbin, China), in September 2018. Then, the seeds and wet sand were mixed (1:2 *v*/*v*), kept in bags, and buried in the experimental nursery of Northeast Forestry University in winter (October–December 2018, about −20 °C) for cold stratification to improve the seed germination for about two months. Subsequently, the sand-mixed seeds were stored in a refrigerator at 4 °C until further use (not more than one month). The seeds were sterilized with 75% alcohol (Lircon, Shandong, China) for 10 s and 2% NaClO (Xilong Scientific, Beijing, China) for 25 min, followed by washing with sterile deionized water, and were then inoculated onto the culture dish lined with wet filter paper (30 seeds per dish). The seeds were allowed to germinate and develop for at least 20 days until the emergence of 2–3 leaves and roots (Figure 1A). Subsequently, the roots of sterile seedlings were removed, the leaves were pruned to about 1 cm, and the hypocotyls were retained as explants (Figure 1B). Additionally, sterile seedlings were cultured in MS medium (Hope Bio-Technology, Qingdao, China) for about 30 days, and then the shoots were removed and the roots were collected. These roots were then cut into 1 cm long segments and used as explants. All the procedures were carried out in sterile conditions. All the cultures were placed in a tissue culture chamber at 25 ± 1 °C under cool-white fluorescent lights (light intensity 25 μmol m^−2^ s^−1^) in 14 h/10 h L/D conditions.

### 4.2. Medium Preparation and Culture Conditions

Standard MS medium with 30 g L^−1^ sucrose was used in all the experiments. The pH of the medium was adjusted to 5.9 (with HCl or NaOH) and the medium was sterilized at 121 °C by autoclaving after adding agar. The medium for callus and adventitious root induction included 7 g L^−1^ agar, and for adventitious shoots 8 g L^−1^ agar was added (Promise Biochemical, Beijing, China). All Petri dishes used in the experiments were sterilized at 121 °C by autoclaving. For callus induction, 30 mL of medium was poured into each sterilized Petri dish, and allowed to cool to solidify, whereas for adventitious shoot and root induction, 60 mL of culture medium was poured into sterilized 330 mL tissue culture bottles and sealed with a vented cap in a sterile environment.

### 4.3. Callus Induction from Explants

When using the hypocotyl as an explant, we removed the roots and upper leaves from sterile seedlings that had been germinated for 20 days, leaving a segment of the embryonic axis approximately 0.5 to 0.8 cm in length as the explant for callus induction. When using sterile root segments as explants, we introduced sterile seedlings into a rooting medium and cultured them for 30 days. Afterward, we cut 2 cm long root segments (while removing thinner adventitious roots from the segments to create wounds), which were then used for callus induction.

Then, we placed the explants, hypocotyls, and root segments horizontally onto the medium supplemented with different combinations of 6-BA (Kulaibo, Beijing, China, 0, 0.5, 1.0, 2.0, or 3.0 mg L^−1^), 2,4-D (Kulaibo, Beijing, China, 0, 0.5, 1.0, 1.5, or 2.0 mg L^−1^), and NAA (Kulaibo, Beijing, China, 0, 0.2, 0.4, 0.6, 1.0, or 2.0 mg L^−1^) for callus induction (Tables S2 and S3). Additional care was taken to ensure that the explants were in full contact with the medium. Each treatment included 20 explants per Petri dish and three such replicates. The sterile seedlings were placed in rooting medium. After rooting for 30 days, relatively coarse adventitious roots were selected and cut into about 2 cm pieces (while cutting off the thinner adventitious roots on the root segment to create wounds). We then inserted them into the medium to induce calli.

### 4.4. Adventitious Shoot Induction

Yellow, granular, healthy calli of almost uniform size were selected and inoculated on the adventitious shoot induction medium. For hypocotyl-induced calli, a medium supplemented with 6-BA (1.0 or 1.5 mg L^−1^) and NAA (0.4 or 1.0 mg L^−1^) in combination with or without 0.5 mg L^−1^ indole-3-butyric acid (IBA, Kulaibo, Beijing, China) was tested (Table S4). For root-induced calli, different combinations of 6-BA (1.0, 1.5, or 2.0 mg L^−1^), NAA (0.4, 0.6, or 0.8 mg L^−1^) and kinetin (KT, Kulaibo, Beijing, China, 0, 0.5, or 1.0 mg L^−1^) were added to the medium (Table S5). After being cultured for 60 days, the induction and appearance of shoots were recorded. Each treatment included 20 calli and three replications.

### 4.5. Root Induction of Adventitious Shoots

We selected the most suitable adventitious bud rooting scheme to induce the growth of robust clustered buds and pruned leaves to 2 cm. Then, they were vertically inserted into the medium supplemented with NAA or IBA to test the rooting effect (Table S7). After 30 days, the percentage (%) of rooting, rooting coefficient (rooting coefficient = total number of roots/number of adventitious buds rooted), and their morphological characteristics were recorded. The number of samples for each treatment was 10 (*n* = 10) and three such replicates were used.

### 4.6. Acclimatization of Plantlets

After 30 days of rooting, the culture bottle caps were removed, and sterile deionized water was added to acclimate the in vitro regenerated plantlets. Three days later, the rooted shoots were removed from the bottles and washed carefully to remove any residual medium from the roots. They were then cultured in sterile water for about 20 days after cleaning the roots and leaves. The rooted shoots were then transplanted into pots filled with peat soil and vermiculite (3:1). The environmental conditions of the plant culture room were 16 h light/8 h darkness and 20–22 °C. The survival of transplanted plants was calculated after 30 days in pots.

### 4.7. Statistical Analysis

Callus induction (%) = the number of induced explants/the number of total inserted explants × 100%

Adventitious shoot induction (%) = the number of calli that induced adventitious shoots/the number of total inserted calli × 100%

Survival (%) = the number of plantlets survived after transplanting/the number of total transplanted plantlets × 100%

Each treatment was repeated three times independently. All data represent the mean value and standard error of mean of three replicates. Data analyses were performed using SPSS 26.0 software. Analysis of variance (ANOVA) was used to determine the significance of the various media combinations. Duncan’s Multiple Range Test was used for pair-wise comparisons of the data.

## 5. Conclusions

**Figure 10:**
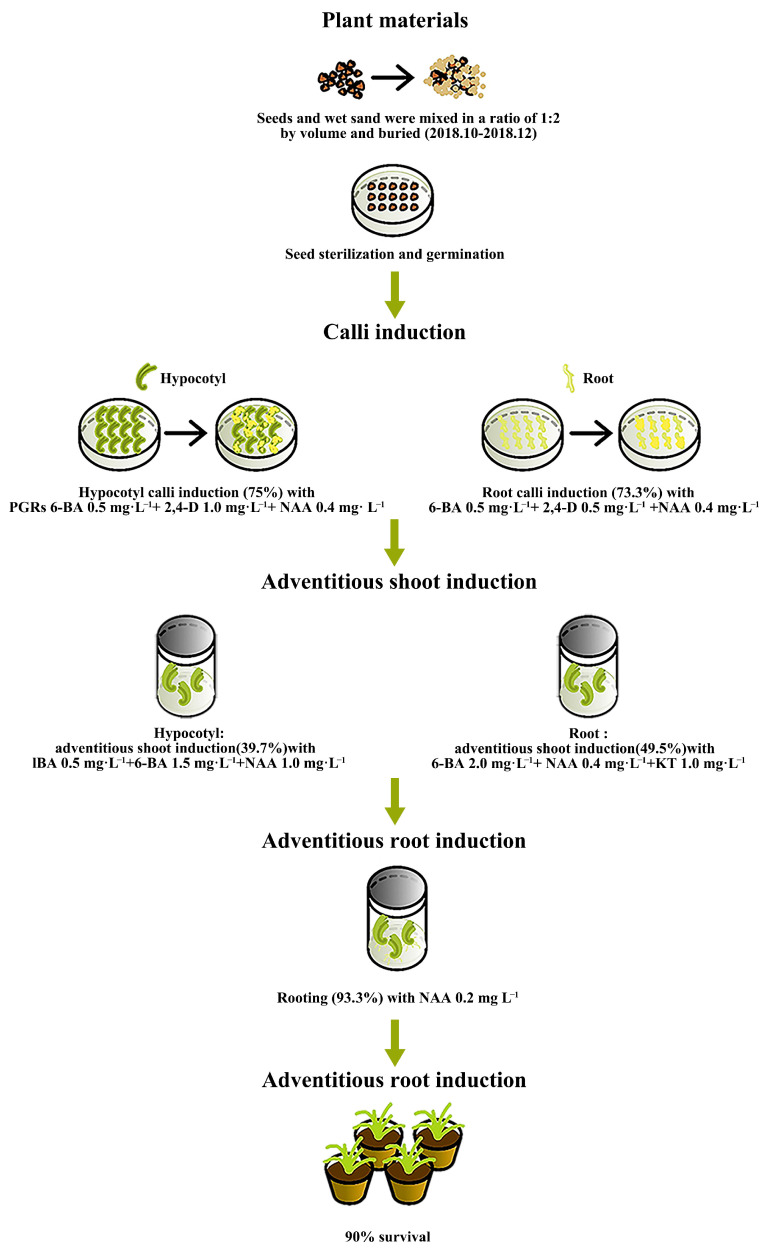
Workflow of the in vitro regeneration system for *I. laevigata*.

This is the first report on the in vitro regeneration system of calli from *I. laevigata*. The design of the entire experimental process is shown in Figure 10. We used two types of explants (hypocotyl and root) for callus induction and obtained the optimal concentration combinations of PGRs that yielded the highest induction rates. The highest hypocotyl callus induction (75.0%) was achieved with PGRs 6-BA 0.5 mg L^−1^ + 2,4-D 1.0 mg L^−1^ + NAA 0.4 mg L^−1^. The highest root callus induction (73.3%) was achieved with 6-BA 0.5 mg L^−1^ + 2,4-D 0.5 mg L^−1^ + NAA 0.4 mg L^−1^. The highest adventitious shoot induction of the hypocotyl induced calli (39.7%) was achieved with IBA 0.5 mg L^−1^ + 6-BA 1.5 mg L^−1^ + NAA 1.0 mg L^−1^; and root induced calli (49.5%) was achieved with 6-BA 2.0 mg L^−1^ + NAA 0.4 mg L^−1^ +KT 1.0 mg L^−1^. Lastly, the best rooting of adventitious shoots reached 93.3% with NAA 0.2 mg L^−1^. The survival of the regenerated plants reached 90.0%. These results will be advantageous for the micropropagation and commercial horticultural application of *I. laevigata* and other *Iris* species.

## Data Availability

All relevant data are contained within the paper.

## Supplementary Materials

The following supporting information can be downloaded at: https://www.mdpi.com/article/10.3390/plants14172733/s1, Table S1: Variance analysis of PGRs influence on the *I. laevigata* hypocotyl callus induction; Table S2: Effect of different PGR concentrations on hypocotyl induced calli and range analysis; Table S3: Effects of different PGRs concentrations on *I. laevigata* root induced calli formation; Table S4: Effects of different PGR combinations on adventitious shoot induction of hypocotyl induced calli; Table S5: Effects of different PGRs combinations on adventitious shoot induction of root induced calli and range analysis; Table S6: Variance analysis of PGRs influence on the adventitious shoot induction of root induced calli; Table S7: Effects of NAA and IBA on root induction of adventitious shoots.
