# Supplementary material for: Establishment of Iris laevigata Tissue Culture Using Hypocotyl and Root Explants
Source: Plants (Basel). 2025 Sep 2;14(17):2733. doi: 10.3390/plants14172733 (PMC12430119; doi:10.3390/plants14172733)
Supplement: Supplementary file 1 [file plants-14-02733-s001.zip › plants-3581575-supplementary.pdf]

Table S1. Variance analysis of PGRs influence on the *I. laevigata* hypocotyl callus induction

Factor	Sum of squares	df	Mean square	F-value	*P*-value
6-BA	11959.259	2	5979.630	20.99	0.000
2,4-D	2603.704	2	1301.852	4.57	0.023
NAA	281.481	2	140.741	0.49	0.620
Error	5698.148	20	284.907	—	—
Total	20542.593	26	—	—	—

Table S2. Effect of different PGR concentrations on hypocotyl induced calli and range analysis

Treatment	Orthogonal array			PGRs (mg L^{-1})			Induction (%)[a, b]	Appearance of calli
	A	B	C	6-BA	2,4-D	NAA		
1	1	1	1	0.5	0.5	0.2	55.00±2.24% bc	yellow, friable, and granular
2	1	2	2	0.5	1.0	0.4	75.00±2.24% a	yellow, friable, and granular
3	1	3	3	0.5	1.5	0.6	43.33±3.33% c	yellow, friable, and granular
4	2	1	2	1.0	0.5	0.4	48.33±4.01% c	yellow, and granular
5	2	2	3	1.0	1.0	0.6	61.67±1.67% b	yellow, lumpy
6	2	3	1	1.0	1.5	0.2	51.67±1.67% bc	yellow, lumpy
7	3	1	3	2.0	0.5	0.6	23.33±6.67% d	yellow, friable, and granular
8	3	2	1	2.0	1.0	0.2	28.33±5.43% d	yellow, friable, and granular
9	3	3	2	2.0	1.5	0.4	21.67±6.54% d	yellow, friable, and granular
[c] K1	—	—	—	57.78	42.22	45	—	—
K2	—	—	—	53.89	55.00	48.33	—	—
K3	—	—	—	24.44	38.89	42.78	—	—
[d] R	—	—	—	33.34	16.11	5.55	—	—

Note: [a] Each value represents the mean ± SEM (standard error of mean) of three independent experiments, and each with 20 hypocotyl explants. [b] Different lowercase letters in the same column indicate significant differences at $P \leq 0.05$ as determined by one-way analysis of variance (ANOVA) with Duncan's post-test. [c] K1, K2, and K3 represent the average induction rates of the same factor at different levels; [d] R means measures of variation, $R = K_{max} - K_{min}$.

The calli originated from the following hormonal combinations:

Treatment 1: 0.5 mg/L 6-BA, 0.5 mg/L 2,4-D, 0.2 mg/L NAA

Treatment 2: 0.5 mg/L 6-BA, 1.0 mg/L 2,4-D, 0.4 mg/L NAA

Treatment 3: 0.5 mg/L 6-BA, 1.5 mg/L 2,4-D, 0.6 mg/L NAA

Treatment 4: 1.0 mg/L 6-BA, 0.5 mg/L 2,4-D, 0.4 mg/L NAA

Treatment 5: 1.0 mg/L 6-BA, 1.0 mg/L 2,4-D, 0.6 mg/L NAA

Treatment 6: 1.0 mg/L 6-BA, 1.5 mg/L 2,4-D, 0.2 mg/L NAA

Treatment 7: 2.0 mg/L 6-BA, 0.5 mg/L 2,4-D, 0.6 mg/L NAA

Treatment 8: 2.0 mg/L 6-BA, 1.0 mg/L 2,4-D, 0.2 mg/L NAA

Treatment 9: 2.0 mg/L 6-BA, 1.5 mg/L 2,4-D, 0.4 mg/L NAA

Table S3. Effects of different PGRs concentrations on *I. laevigata* root induced calli formation

Treatment	6-BA (mg L^{-1})	2,4-D (mg L^{-1})	NAA (mg L^{-1})	Induction (%)[a, b]	Appearance of Calli
1	0.0	0.5	0.4	0.00±0.00% d	No calli
2	0.5	0.5	0.4	73.33±9.89% a	yellow, small
3	2.0	0.5	0.4	66.67±12.29% a	yellow, small
4	3.0	0.5	0.4	26.67±4.22% bc	yellow, small
5	0.5	0.0	0.4	6.67±4.22% d	yellow, small
6	0.5	1.0	0.4	36.67±3.33% b	yellow, small
7	0.5	2.0	0.4	16.67±3.33% cd	yellow, small
8	0.5	0.5	0.0	3.33±3.33% d	yellow, small
9	0.5	0.5	1.0	30.00±4.47% bc	yellow, small
10	0.5	0.5	2.0	13.33±4.21% cd	yellow, small

Note: [a] Each value represents the mean ± SEM of three independent experiments, and each with 20 explants. [b] Different lowercase letters in the same column indicated the significant difference at $P \leq 0.05$ as determined by one-way analysis of variance (ANOVA) with Duncan's post-test.

Table S4. Effects of different PGR combinations on adventitious shoot induction of hypocotyl induced calli

Treatment	IBA(mg L^{-1})	6-BA (mg L^{-1})	NAA (mg L^{-1})	Adventitious shoot induction (%)[a, b]	Appearance of adventitious shoots
1	0.0	1.0	0.4	00.00±0.00% c	calli browning
2	0.5	1.0	0.4	16.67±8.33% b	green, thick
3	0.5	1.5	1.0	39.72±15.28% a	green, thick

Note: [a] Each value represents the mean ± SEM of three independent experiments, and each with 20 hypocotyl explants. [b] Different lowercase letters in the same column indicated the significant difference at $P \leq 0.05$ as determined by one-way analysis of variance (ANOVA) with Duncan's post-test.

The callus originated from the following hormonal combinations:

Treatment 1: 0.0 mg L^{-1} IBA, 1.0 mg L^{-1} 6-BA, 0.4 mg L^{-1} NAA.

Treatment 2: 0.5 mg L^{-1} IBA, 1.0 mg L^{-1} 6-BA, 0.4 mg L^{-1} NAA.

Treatment 3: 0.5 mg L^{-1} IBA, 1.5 mg L^{-1} 6-BA, 1.0 mg L^{-1} NAA.

Table S5. Effects of different PGRs combinations on adventitious shoot induction of root induced calli and range analysis

Treatment	Orthogonal array			PGRs (mg L^{-1})			Adventitious shoot induction (%)[a, b]	Appearance of adventitious shoots
	A	B	C	6-BA	NAA	KT		
1	1	1	1	1.0	0.4	0.0	7.50±4.79% c	greenish yellow, slender
2	1	2	2	1.0	0.6	0.5	15.72±6.12% bc	greenish yellow, slender
3	1	3	3	1.0	0.8	1.0	23.33±3.33% bc	deep green, thick
4	2	1	2	1.5	0.4	0.5	16.67±8.03% bc	green, thick
5	2	2	3	1.5	0.6	1.0	26.67±4.22% bc	green, thick
6	2	3	1	1.5	0.8	0.0	13.33±4.22% bc	green, thick
7	3	1	3	2.0	0.4	1.0	49.52±11.13% a	deep green, thick
8	3	2	1	2.0	0.6	0.0	26.67±4.22% bc	deep green, thick
9	3	3	2	2.0	0.8	0.5	30.00±4.47% b	green, slender
[c]K1	—	—	—	15.52	24.56	15.83	—	—
K2	—	—	—	18.89	23.02	20.80	—	—
K3	—	—	—	35.40	22.22	33.17	—	—
[d]R	—	—	—	19.88	2.34	17.34	—	—

Note: [a] Each value represents the mean ± SEM of three independent experiments, and each with 20 root induced calli. [b] Different lowercase letters in the same column indicated the significant difference at $P \leq 0.05$ as determined by one-way analysis of variance (ANOVA) with Duncan's post-test. [c] K1, K2, and K3 represent the average induction rates of the same factor at different levels; [d] R means measures of variation, $R = K_{max} - K_{min}$.

The callus originated from the following hormonal combinations:

Treatment 1: 1.0 mg/L 6-BA, 0.4 mg/L NAA, 0.0 mg/L KT

Treatment 2: 1.0 mg/L 6-BA, 0.6 mg/L NAA, 0.5 mg/L KT

Treatment 3: 1.0 mg/L 6-BA, 0.8 mg/L NAA, 1.0 mg/L KT

Treatment 4: 1.5 mg/L 6-BA, 0.4 mg/L NAA, 0.5 mg/L KT

Treatment 5: 1.5 mg/L 6-BA, 0.6 mg/L NAA, 1.0 mg/L KT

Treatment 6: 1.5 mg/L 6-BA, 0.8 mg/L NAA, 0.0 mg/L KT

Treatment 7: 2.0 mg/L 6-BA, 0.4 mg/L NAA, 1.0 mg/L KT

Treatment 8: 2.0 mg/L 6-BA, 0.6 mg/L NAA, 0.0 mg/L KT

Treatment 9: 2.0 mg/L 6-BA, 0.8 mg/L NAA, 0.5 mg/L KT

Table S6. Variance analysis of PGRs influence on the adventitious shoot induction of root induced calli

Factor	Sum of squares	df	Mean square	F-value	*P*-value
6-BA	4074.713	2	2037.357	3.98	0.036
NAA	51.030	2	25.515	0.05	0.890
KT	3571.062	2	1785.531	3.49	0.043
Error	10230.719	20	511.536	—	—
Total	17228.058	26	—	—	—

Note: Each value is determined by three-way analysis of variance (ANOVA) with Duncan's post-test.

Table S7. Effects of NAA and IBA on root induction of adventitious shoots

Treatment	NAA (mg L^{-1})	IBA (mg L^{-1})	Rooting (%)[a,b]	Rooting coefficient	Appearance of roots
1	0.2	—	93.33±6.67% a	7.17±0.33 b	yellow, thick, and fast growing
2	0.5	—	93.33±4.44% a	6.22±0.20 c	greenish yellow, thick
3	1.0	—	83.33±7.45% a	8.12±0.38 a	yellow, thick, and slow growing
4	—	0.2	96.67±3.33% a	3.38±0.20 e	greenish yellow, slender
5	—	0.5	86.67±5.44% a	3.13±0.30 e	yellow, slender
6	—	1.0	90.00±5.09% a	4.58±0.35 d	greenish yellow, slender

Note: [a] Each value represents the mean ± SEM of three independent experiments, and each with 20 adventitious shoots. [b] Different lowercase letters in the same column indicated the significant difference at $P \leq 0.05$ as determined by one-way analysis of variance (ANOVA) with Duncan's post-test.

The shoots originated from the following hormonal combinations:

Treatment 1: 0.2 mg/L NAA

Treatment 2: 0.5 mg/L NAA

Treatment 3: 1.0 mg/L NAA

Treatment 4: 0.2 mg/L IBA

Treatment 5: 0.5 mg/L IBA

Treatment 6: 1.0 mg/L IBA
